# Cyclic AMP induction of *Dictyostelium* prespore gene expression requires autophagy

**DOI:** 10.1016/j.ydbio.2019.04.017

**Published:** 2019-08-15

**Authors:** Yoko Yamada, Pauline Schaap

**Affiliations:** School of Life Sciences, University of Dundee, DD15EH, Dundee, UK

**Keywords:** Cell-type specialization, Sporulation, Autophagy, atg7, atg5, atg9, cAMP receptors

## Abstract

*Dictyostelium discoideum* amoebas display colonial multicellularity where starving amoebas aggregate to form migrating slugs and fruiting bodies consisting of spores and three supporting cell types. To resolve the cell signalling mechanism that control sporulation, we use insertional mutagenesis of amoebas transformed with fusion constructs of spore genes and red fluorescent protein. We identified the defective gene in a mutant lacking spore gene expression as the autophagy gene Atg7. Directed knock-out of *atg7* and of autophagy genes like *atg5* and *atg9* yielded a similar phenotype, with lack of viable spores and excessive differentiation of stalk cells. The *atg7-*, *atg5-* and *atg9-* cells were specifically defective in cAMP induction of prespore genes, but showed enhanced cAMP stimulation of prestalk genes at the same developmental stage. The lack of prespore gene induction in the autophagy mutants was not due to deleterious effects of loss of autophagy on known components of the cAMP pathway, such as cAMP receptors and their cAMP-induced phosphorylation and internalization, PKA and the transcription factors SpaA and GbfA, or to lack of NH_3_ production by proteolysis, which was previously suggested to stimulate the spore pathway. Our continued mutagenesis approach is the most likely to yield the intriguing link between autophagy and prespore gene induction.

## Highlights

• A screen for *Dictyostelium* sporulation mutants identified autophagy gene *atg7*.
• *Atg7-*, *atg5-*and *atg9-*cells cannot form viable spores, but show normal stalk formation.
• *Atg7-*, *atg5-*and *atg9-*are specifically defective in cAMP induction of prespore genes.
• *Atg7-*, *atg5-*and *atg9-*show normal to enhanced cAMP induction of prestalk genes.
• PKA overexpression does not restore sporulation in autophagy mutants.

## 1 Introduction

Autophagy is an ancient survival strategy that allows eukaryotic cells to survive starvation by enclosing and digesting cytosolic components and organelles. A large number of genes required for autophagy were initially identified in yeast and many proved to be deeply conserved in animals, plants and other eukaryotes. At the structural level, autophagy initiates with the formation of crescent-shaped double-membraned structures called phagophores, which enclose cellular contents and fuse at their termini to form an autophagosome vesicle. The autophagosome subsequently fuses with a primary lysosome to form an autolysosome where both the inner membrane and captured cargo are degraded and the catabolites are fed back into the cell by integral membrane permeases. At the molecular level, autophagy initiates when amino acid starvation blocks phosphorylation of Atg13 by the Target of Rapamycin C1 (TORC1) kinase, which prevents Atg13 from forming a complex with Atgs 1, 17, 29 and 31 and to initiate a phagophore assembly site (PAS). The phosphatidylinositol 3-kinase (PtdIns3K) complex consisting of Vps34, Vps15, Atg6, Atg14 and Atg38 generates PIP3 at the PAS, which recruits Atg18 and Atg2 to the PAS and in turn Atg9, Atg8 and Atg12. The transmembrane protein Atg9 and its associates Atgs11, 23 and 27 direct membrane to the PAS to cause phagophore expansion. Two ubiquitin-like conjugation systems composed of Atgs 5, 7, 10, 12 and 16 and Atgs 3, 4, 7 and 8 further regulate vesicle expansion (Feng et al., 2014; Mizushima et al., 2011; Yin et al., 2016).

Autophagy is also important for the multicellular life cycle of *Dictyostelium* amoebas, which survive starvation by aggregating to form fruiting bodies with dormant spores and dead stalk cells. Autolysosomes appear within 2 h of starvation and increase in number during aggregation. Thereafter, autolysomes become less prominent in the presumptive spore cells and more numerous in the prestalk cells, where they finally fuse to form the plant-like vacuole of the stalk cells (Schaap, 1983; Schaap et al., 1981; Uchikawa et al., 2011). As a genetic model *D. discoideum* is particularly suitable for identification of essential components of the autophagy pathway, and up to date the roles of many components were identified, such as Atgs 1 and 13 of the Atg1 complex, Vps34, Vps15 and Atg14 of the PtdIns3K complex, Atgs 2 and 18 of the PIP3 binding complex, Atg9 of the membrane trafficking system, Atgs 5, 7, 10, 12 and 16/tipD of one and Atgs 3, 4, 7 and 8 of the other ubiquitin-like conjugation complex, as well as a homolog of the mammalian autophagy gene Atg101, a member of the Atg1 complex (Calvo-Garrido et al., 2010; Mesquita et al., 2017). Studies in *Dictyostelium* identified a role for Vmp1 and Vps13A/tipC in autophagy (Muñoz-Braceras et al., 2015) and revealed novel autophagy genes such as the autophagy inhibitors *areA* and *areB* (Mesquita et al., 2015).

Deletion of most of these genes prevent autophagosome formation and block autophagy mediated proteolysis. Lesions in *atg1*, *atg13* and *vmp1* yield the most severe phenotype with cells failing to aggregate upon starvation and, for *atg1-* and *vmp1-*cells*,* to differentiate into stalk cells *in vitro*. Deletions of other autophagy genes, such *atgs 5, 7, 8, 9, 16/tipD, 101 and vps13A/tipC* yield amoebas that can aggregate, but thereafter show delayed and abnormal development. Instead of one sorogen or slug, the aggregate gives rise to multiple small ones, which eventually turn into fruiting bodies with abnormal spores (Calvo-Garrido et al., 2010; Mesquita et al., 2017).

We investigate the signalling pathways that control prespore and spore differentiation and use mutagenesis of amoebas transformed with an mRFP tagged spore coat gene to identify pathway components. We recovered a mutant defective in sporulation, but overproducing stalk cells. The genetic lesion occurred in the *atg7* gene and further analysis of a re-created *atg7* knockout and knock-outs in *atg5* and *atg9* revealed that these genes were specifically required for induction of prespore gene expression, but dispensable for stalk cell differentiation *in vivo* and *in vitro*.

## 2 Materials and methods

### 2.1 Cell culture

*Dictyostelium discoideum* Ax2 was cultured either in HL5 axenic medium (Formedium, UK) or on SM agar plates in association with *Klebsiella aerogenes*. For development, cells were distributed at 2.5 × 10^6^ cells/cm^2^ on non-nutrient agar and for β-galactosidase staining on dialysis membrane supported by non-nutrient agar. *atg9-* cells (Tung et al., 2010) were obtained from the Dicty stock center http://dictybase.org/StockCenter/StockCenter.html.

### 2.2 Knock-out and expression constructs

To generate an *atg7* knock-out vector, 5′ and 3′ fragments of the genomic region containing the *atg7* gene were amplified using primer pairs atg7-5′f/atg7-5′r and atg7-3′f/atg7-3′r (Table 1), respectively, subcloned into vector pJet1.2blunt, and cloned into plasmid pLPBLP (Faix et al., 2004) using pstI/BamHI for the 5′ fragment and HindIII/SalI for the 3′ fragment respectively. The pLPBLP–atg7KO vector was linearised with ScaI and transformed into Ax2 cells.

**Table 1:** Oligonucleotide primers used for plasmid constructs.

atg5-5′f	ggatccCGTACCAATCGATTCAACTC
atg5-5′r	ctgcagCACCTATAGGTAAATGCCAC
atg5-3′f	aagcttCCCAATTCAAGAACTAATACCAG
atg5-3′r	gtcgacCCACAACCACTACTGCAAC
atg5KO1	CAATCAAATGATCTTTGGGATGG
atg5KO2	CAGGTTCAGCTGATTCCAC
atg5KO3	CTGGTTGAAGGTTTCGATAGAC
atg7-5′f	ggatccGACGAAACGACTTATAGTCC
atg7-5′r	ctgcagGTTGAATTGGTTGTGATGG
atg7-3′f	aagcttGGGTTTCGACTCTTATCTAG
atg7-3′r	gtcgacGGTCCAAGCACGTGAGGG
atg7KO1	GCCAGGTCATTCCGTACCTC
atg7KO2	GATGGCATAACTCTCCATCTC
atg7KO3	CTGTAGGCTCAAATGCTGAAG
Bsr-r	GCCGCTCCCACATGATG
Bsr-f	GTGGTAAGTCCTTGTGG
atg7-f	gaattcATGACAAATACACTTCAGTTTAAAG
atg7-r	atcgatATCATCAGAAATATCAATATCCC
atg7_mf	GATCAAATGGCTACCGTTACTAGAC
atg7_mr	CTAGTAACGGTAGCCATTTGATCTAAAG
YFP-f	CCGACAACCACTACCTGAGCTA
2Hterm-r	GGATCACTTGATTCTTCATCGGATC

**Fig. 1:**
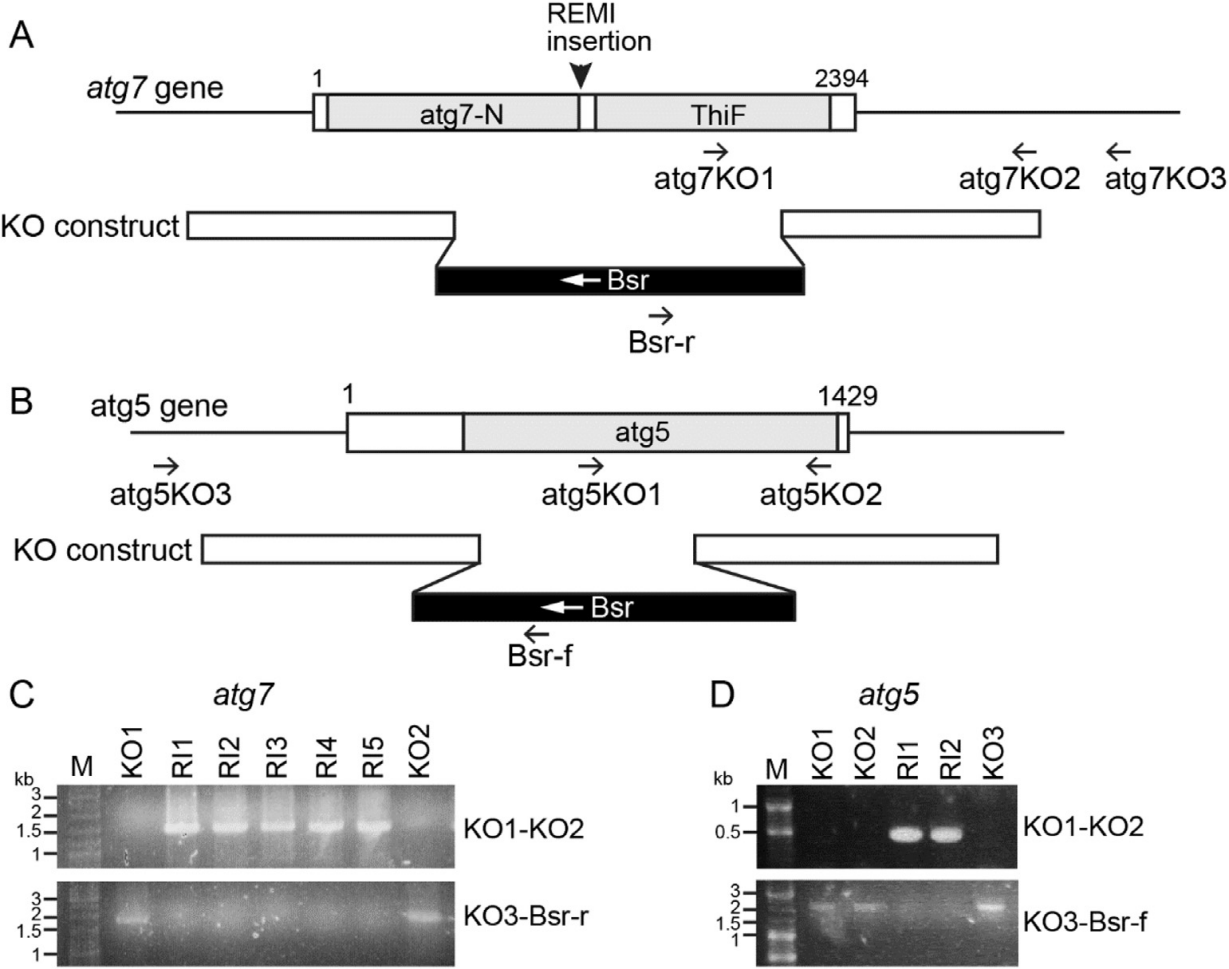
**Knockout of *atg7* and *atg5* genes**. *A, B. Constructs*. Diagram of the *atg7* (A) and *atg5* (B) genes and their knockout constructs with the blasticidin resistant cassette (Bsr). Arrows indicate the position of the primers used in verification of the knockout clones. *C, D. Diagnosis.* Genomic DNA was prepared from clonal isolates of Ax2 transformed with the knockout construct for *atg7* (C) or *atg5* (D), and analysed by PCR. Primer pair atg7KO1 and KO2 amplifies a 1.5 kb fragment from random integrants only, whereas primer pair atg7KO3 and Bsr-r amplifies a 1.9 kb fragment from knockout clones (C). Primer pair atg5KO1 and KO2 amplifies a 0.5 kb fragment from random integrants, while primer pair atg5KO3 and Bsr-f amplifies a 2 kb fragment from knockout clones (D).

To generate an *atg5* knock-out vector, 5′ and 3′ fragments of genomic region of the *atg5* gene was amplified using primer pairs atg5-5′f/atg5-5′r and atg5-3′f/atg5-3′r (Table 1), subcloned into pJet1.2blunt and cloned into pLPBLP using pstI/BamHI and HindIII/SalI digestion. The BamHI/SalI fragment was excised from the vector and transformed into Ax2. Transformants were selected at 10 μg/ml blasticidin and diagnosed for *atg7* or *atg5* gene disruption by two PCR reactions (Fig. 1).

To generate wild-type and mutant *atg7* expression constructs, the act15p-YFP fragment of pDd-NYFP was cloned into pExp4-Hyg (Yamada et al., 2018) using SalI/XhoI to generate Dd-NYFP-Hyg. The atg7 coding region was amplified from genomic DNA of Ax2 with primers atg7-f and atg7-r (Table 1), subcloned into pJet1.2blunt, and cloned into Dd-NYFP-Hyg with EcoRI/ClaI to create act15p-YFP-atg7. To generate a Cys563 to Ala point mutation, 5′ and 3′ *atg7* fragments were amplified from act15p-YFP-atg7 using primers YFP-f and atg7-mr and atg7-mf and 2Hterm-r (Table 1). After annealing the fragments, atg7Cys563Ala was amplified with atg7-f and atg7-r and cloned into Dd-NYFP-Hyg using EcoRI/ClaI. Constructs were transformed into *atg7-* cells by electroporation and transformants were selected at 30 μg/ml hygromycin.

### 2.3 Western analysis of expressed proteins

Cells were lysed in SDS-sample buffer, proteins were separated on 4–12% polyacrylamide gels (Thermo Fisher Scientific, Whaltham, MA), transferred to nitrocellulose and probed with anti-GFP antibody (Roche Applied Science, Penzberg, Germany), followed by HRP-conjugated anti-mouse antibody. YFP-positive bands were detected using SuperSignal West Pico Chemiluminescent Substrate (Thermo Fisher Scientific, Whaltham, MA).

### 2.4 *In vitro* induction of stalk cell differentiation

Cells harvested from growth medium were resuspended in stalk salts (10 mM MES buffer pH 6.2, 10 mM KCl, 2 mM NaCl, 1 mM CaCl_2_) at 2 × 10^5^ cells/ml and distributed as 1.25 ml aliquots in a 6-well culture dish. After 2 h at 22 °C, medium was supplemented with 1 mM cAMP, and after further incubation of 6–7 h, the medium was replaced with stalk salts containing 100 nM DIF-1 (Enzo biochem, New york). After further incubation for 16–23 h, vacuolation of the cells was examined by phase contrast microscopy.

### 2.5 Induction of developmental gene expression

For induction of prespore genes, cells were developed on non-nutrient agar at 12 °C overnight and then at 22 °C for a few hours until loose mounds had formed. Mounds were then dissociated, resuspended to 5 × 10^6^ cells/ml in 1 mM MgCl_2_ in KK2 (20 mM potassium phosphate buffer, pH 6.2) and incubated in the presence and absence of 1 mM cAMP. For induction of *ecmA*, dissociated loose mound cells were resuspended in stalk salts at 3 × 10^6^ cells/ml and shaken with or without 1 mM cAMP and 100 nM DIF-1. For induction of *cprB*, cells were starved at 4 °C overnight to induce aggregation competence and then shaken at 3 × 10^6^ cells/ml in KK2 with or without 1 mM cAMP. For all genes, cells were incubated with cAMP and/or DIF-1 for 4 h at 22 °C and subsequently harvested for RNA isolation. The transcript levels of the different genes were analysed by RT-qPCR using the primers listed in Table 2 as described previously (Yamada et al., 2018).

**Table 2:** Oligonucleotide primers used for qRT-PCR.

cotC-f	GAAAGACGTGGTGGTATC
cotC-r	TTGCATCTTGGAAGTCATC
pspA-f	GCACTCGGTTCTGATTGGAG
pspA-r	GATGTTTGGGATGGGGTTGG
cprB-f	GAGAATGGTGCTCAACATGG
cprB-r	CTGGACCTTCATCATCTTTACC
ecmA-f	CCGTAAACTGTGAATGTGATGACC
ecmA-r	GTCTTGGAATCGCAACTATCAGC
spaA-f	CACCAGGATCAACAATGGG
spaA-r	AACGGTCGGTAAGGATATCG
gbfA-f	TCAACCTCTGTATCATGTCC
gbfA-r	ATGGTGAAAGTCCTGCACC
Ig7-f	AACAGCTATCACCAAGCTTGATTAGCC
Ig7-r	TTACATTTATTAGACCCGAAACCAAGCG
carA-f	GGATCCGGTCTTTTAGATGGAAATCCAG
carA-r	TCAACACTGCCATACAACCC

To induce and assay cotC-gal, cells from dissociated loose mounds were resuspended at 3 × 10^6^ cells/ml in 1 mM MgCl_2_ in KK2 and shaken for 6 h as 80 μl aliquots in microtiter plates. Cells were lysed by freeze-thawing, supplemented with 20 μl of 5x assay buffer (500 mM NaH_2_PO_4_/Na_2_HPO pH 7, 50 mM KCl, 5 mM MgSO_4_, 2 mM MgCl_2_, 2% β-mercaptoethanol and 5 mM chlorophenol red-β-D-galactopyranoside) and incubated at room temperature. OD574 was measured at regular intervals using a microtiter plate reader.

Visualization of β-galactosidase expression in intact structures was performed using established procedures (Dingermann et al., 1989).

## 3 Results

### 3.1 Lesion of *Atg7* impairs spore but not stalk differentiation in *D. discoideum*

**Fig. 2:**
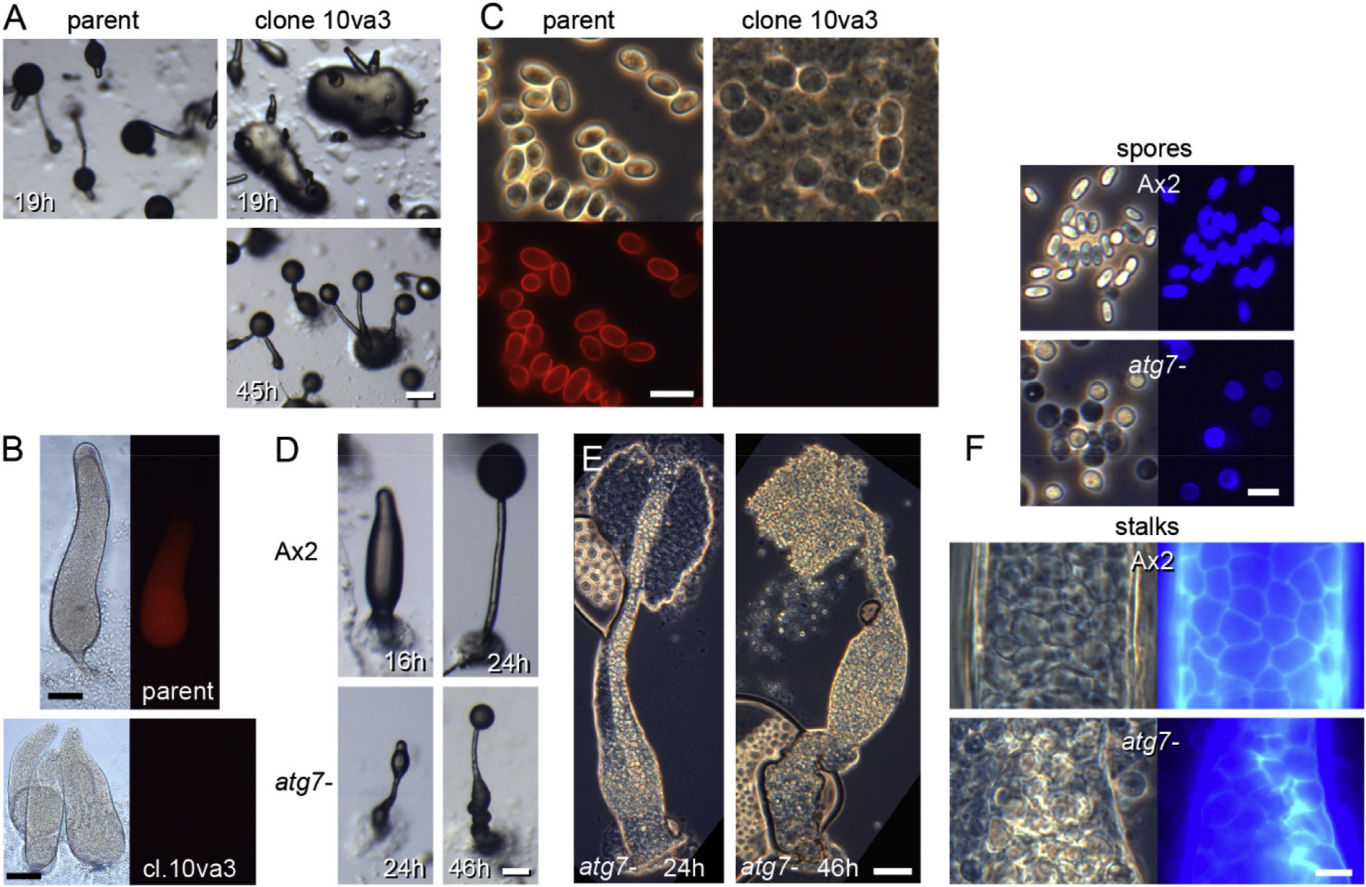
**Identification of atg7 by REMI mutagenesis and validation by gene knock-out**. *A-C. Phenotype of parental strain Ax2/cotC-mRFP and REMI clone 10va3.* A. Developing structures at 19 h and 45 h. Bar: 200 μm. B. Sorogens were photographed under phase contrast and epifluorescence. Bar: 100 μm. C. Spores of terminal structures under phase contrast (top) and epifluorescence (bottom) illumination. Bar: 10 μm. *D-F. Phenotype of the recapitulated atg7 knockout.* Developing Ax2 and *atg7-* structures were photographed *in situ* (D) at 16, 24 and 46 h, or in case of *atg7-* also squashed under a coverslip (E). Bar: 50 μm. F. Spores and stalks from mature Ax2 and *atg7-* fruiting structures, stained with 0.002% Calcofluor and photographed under phase contrast and epifluorescence. Bar 10 μm.

In order to identify genes that control *Dictyostelium* sporulation, we performed REMI mutagenesis on Ax2 cells transformed with a fusion construct of mRFP and the spore coat gene *cotC*, expressed from its own promoter (Yamada et al., 2018) and screened for mutants with spore defects. CotC-mRFP is localized to Golgi-derived prespore vesicles in prespore cells and is exocytosed during fruiting body formation to be incorporated into the spore wall. We isolated a clone 10va3, which failed to form spores. The cells were slightly (∼1 h) delayed in aggregation and more strongly in post-aggregative development, forming mounds with multiple early sorogens (a.k.a. first fingers) at 19 h, when the parental cells had nearly completed fruiting body formation (Fig. 2A). Expression of cotC-mRFP was undetectable in sorogens (Fig. 2B), suggesting that prespore differentiation is altered. The 10va3 sorogens eventually formed small fruiting bodies at 45 h on top of a basal cell mass, but cells in their spore heads were mostly round rather than elliptical as is the case for spores, and were not labelled with cotC-mRFP (Fig. 2C). Stalks contained vacuolated cells and thus stalk cell differentiation did not seem to be disturbed.

Sequencing of the genomic region flanking the inserted plasmid showed that the insertion in clone 10va3 occurred at a BamHI site in the *atg7* gene. Knock-out of the *atg7* gene was reported previously to result in loss of autophagy, formation of multi-tipped aggregates and defective spore differentiation (Otto et al., 2003), but a role for Atg7 in prespore gene expression was not reported. In addition, the formation of seemingly normal stalks in 10va3 was unexpected, since the autophagy gene *atg1* is essential for stalk cell differentiation *in vitro* (Kosta et al., 2004). To analyse the role of Atg7 in cell differentiation in more detail, we created an *atg7* knock-out by deleting a central region of the gene that contained about half of its Atg7-N and ThiF domains (Fig. 1A,C).

The *atg7-* cells recapitulated the phenotype of clone 10va3 (Fig. 2D–F). Development was delayed several hours compared to parental Ax2 cells, but *atg7-* cells eventually formed small fruiting bodies with a thickened lower part. In contrast to the elliptical phase bright spores of Ax2, cells in *atg7-* spore heads were round and often phase dark. Most of these cells were not stained with calcofluor, a cellulose binding dye, although a fraction showed weak staining (Fig. 2F). Quantitation showed that only 6% of *atg7-* cells produced detergent resistant spores, of which 1/3rd germinated to yield viable amoebas (Table 3). Malformation of the fruiting body and poor sporulation are consistent with the previously described insertion mutant of *atg7-* (Otto et al., 2003). We also confirmed that spore defects are cell-autonomous, since the *atg7-* cells did not form viable spores when mixed with Ax2 (Table 3).

**Table 3:** Spore production in *atg7-* cells.

strain	time (h)	detergent resistant cells (%)^a^	germinating spores (%)^b^	overall viable spores (%)^c^
Ax2	25	149 ± 4	85 ± 7	127 ± 13
*atg7-*	43–49	6 ± 6	32 ± 31	2 ± 1
1:1 Ax2/*atg7-*	25	89 ± 50	76 ± 19 (All Ax2)^d^	

Ax2, *atg7-* and a 1:1 mixture of AX2 and *atg7-* cells were plated on 1 cm^b^ nitrocellulose filters supported by NN agar at 2.5 × 10^6^ cells per filter. Filters were vortexed with 0.1% Triton-X100 when fruiting bodies had formed.

a Detergent resistant spores were counted and data are expressed as percentage of the plated cell number. Ax2 cells show >100% spores due to some cell division occurring during development.

b The detergent resistant spores were clonally plated on bacterial lawns, after 2–5 days emerging plaques were counted and expressed as percentage of the plated spores.

c The overall percentage of viable spores was determined as (fraction of triton-resistant x fraction of germinated spores) x 100.

d The genotype of the germinated spores was evaluated from the developmental phenotype.

Similar to clone 10va3, the *atg7-* fruiting bodies formed a stalk that penetrated the (pre)spore cell mass and connected to the basal cell mass (Fig. 2E and F). The cells inside the stalk were highly vacuolated and encapsulated in cellulose, although arrangement of the *atg7-* stalk cells was somewhat irregular compared to wild type. The cells in the expanded bottom region of the stalk eventually also vacuolated. These results show that Atg7 is essential for sporulation, while it is dispensable for stalk cell differentiation.

**Fig. 3:**
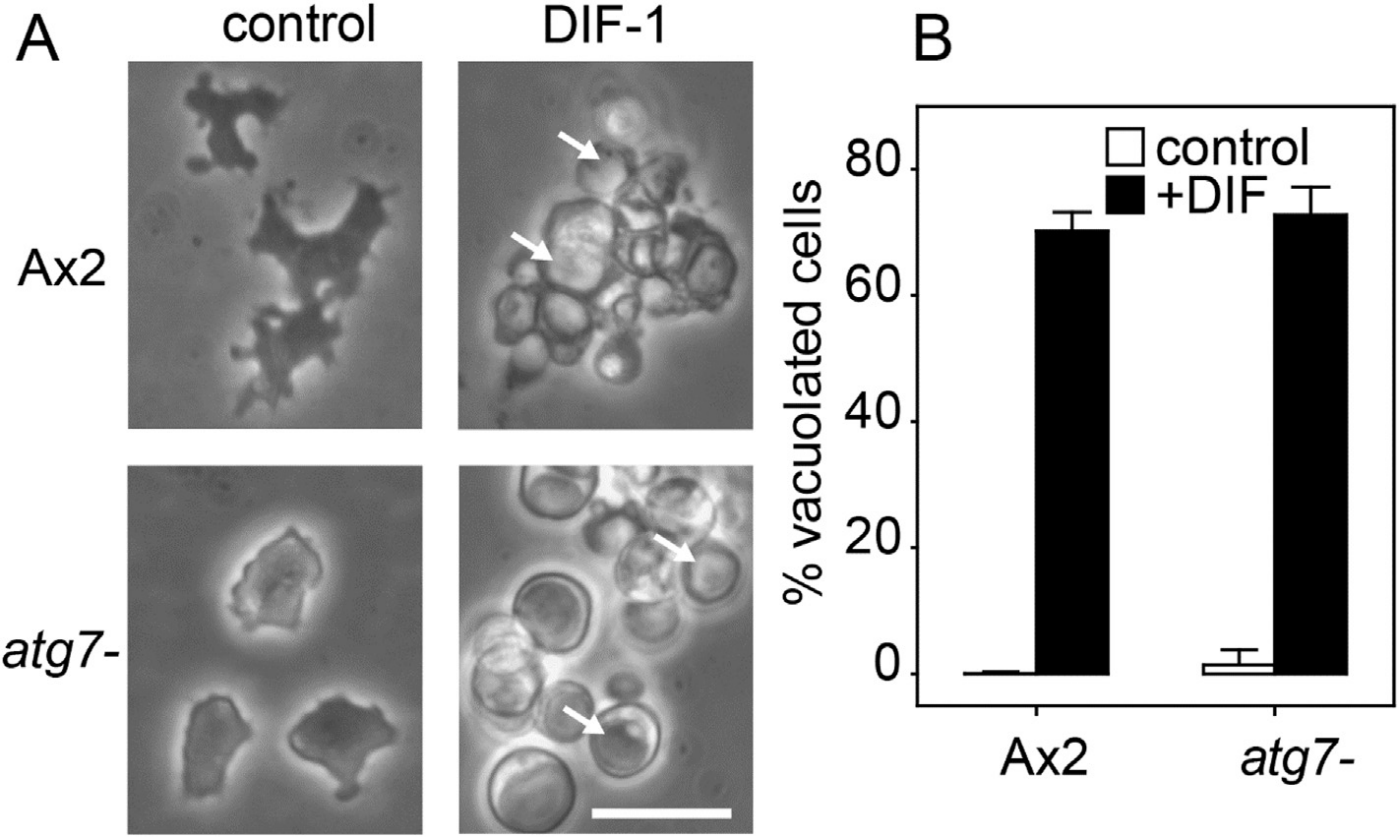
**Stalk cell induction *in vitro***. Ax2 and *atg7-* cells were pre-incubated for 6 h with 1 mM cAMP. After removal of cAMP, cells were incubated for 23 h with 100 nM DIF-1. Control cells received the DIF-1 solvent, 0.1% ethanol. About 70 cells per sample were photographed, with representative images shown in (A). Some of the typical stalk cell vacuoles are indicated by arrows. Bar: 20 μm. B. Percentages of vacuolated over total cells were determined from images. Means and SD of three experiments. Values for DIF-treated cells were not statistically different between Ax2 and *atg7-* (*t*-test, P = 0.46).

The earlier reports describing requirement for the autophagy protein Atg1 in stalk cell differentiation were based on cells differentiating in a monolayer in the presence of the polyketide DIF-1 (Kosta et al., 2004). To test whether Atg7 is required under these conditions, we rendered *atg7-* cells in monolayers competent to DIF-1 by pre-incubation with cAMP (Berks and Kay, 1990) and then stimulated cells with DIF-1. In contrast to *atg1-* cells (Kosta et al., 2004), *atg7-* and Ax2 cells readily vacuolated in response to DIF-1 (Fig. 3), although most vacuoles of the *atg7-* cells appeared to contain more material than those of Ax2. Both strains remained amoeboid in the absence of DIF-1. Apparently, Atg7 is not required for DIF-induced stalk cell vacuolation *in vitro*.

### 3.2 Expression of cell type markers in *atg7*-

The lack of cotC-mRFP expression in 10va3 sorogens (Fig. 2B) suggests that the abolished spore production is due to defective prespore differentiation. To examine cell differentiation in more detail, we transformed Ax2 and *atg7-* cells with fusion constructs of cell type specific promoters and the *LacZ* reporter gene (gal). The transformants were developed to early and late sorogens and fruiting bodies, and stained with X-gal to visualize β-galactosidase expression.

**Fig. 4:**
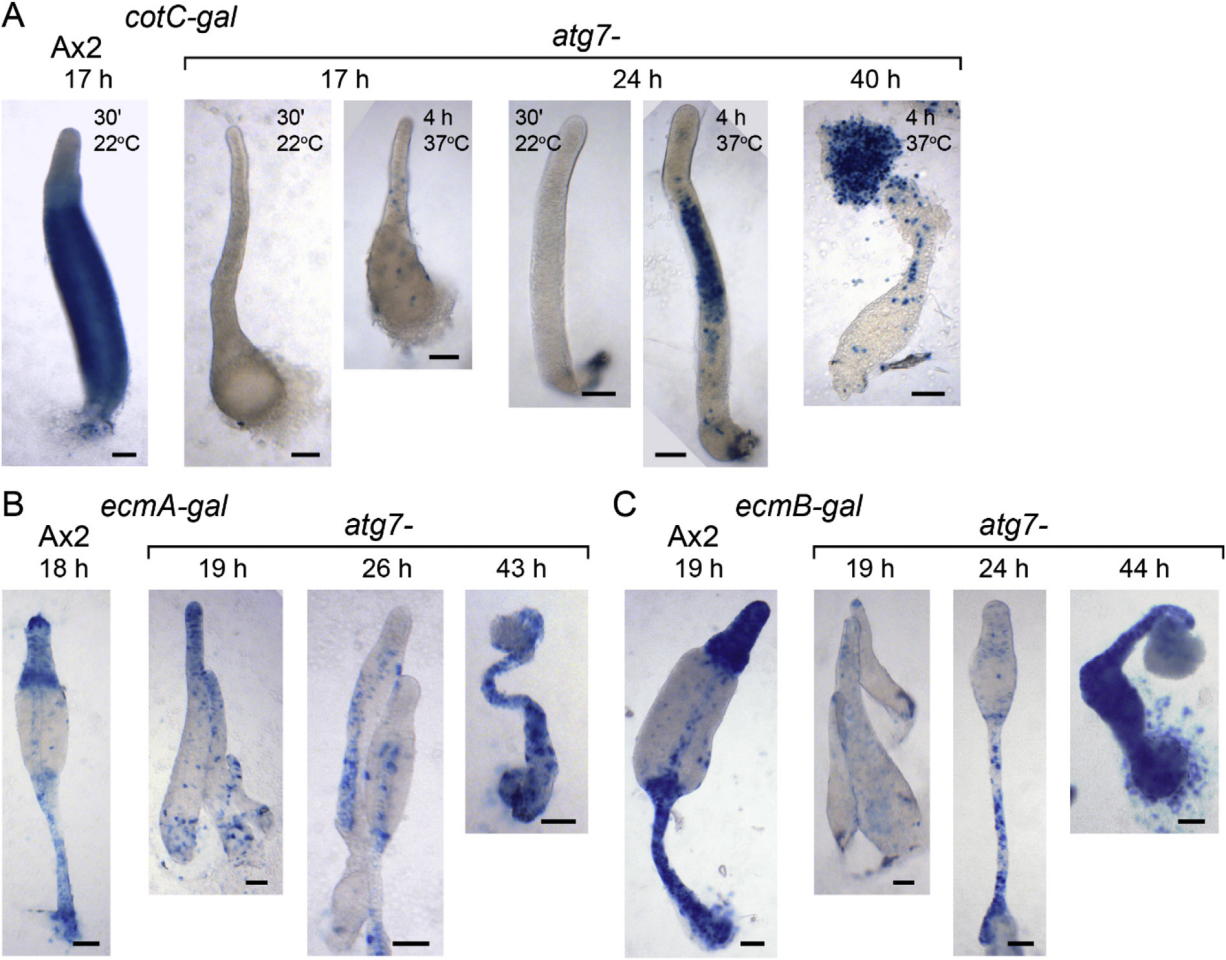
**Expression pattern of cell-type markers in *atg7-***. Ax2 and *atg7-* cells, transformed with *cotC-gal* (A), *ecmA-gal* (B) or *ecmB-gal* (C) were incubated on dialysis membrane supported by non-nutrient agar for the time periods shown above the images. Structures were fixed and stained with X-gal for 30 min at 22 °C for all structures, and also for 4 h at 37 °C for *atg7-/cotC-gal* structures as shown inside the images. Bar: 50 μm.

The prespore marker *cotC-gal* is expressed strongly in the posterior prespore region of Ax2 early culminants at 17 h, but at 17 and 24 h expression of *cotC-gal* in *atg7-* was very low and only detectable after prolonged staining with X-gal (Fig. 4A). At 24 h, *cotC-gal* expression was still very weak and confined to a short central region of the sorogen. In *atg7-* fruiting bodies, some *cotC-gal* expression was detected in the abnormal spores of the spherical heads. The prestalk markers *ecmA-gal* and *ecmB-gal* were expressed in the primary stalks of *atg7-* fruiting structures at about the same levels as in Ax2 cells. The vacuolating cell masses at the base of the *atg7-* stalks only started to express the prestalk markers very late in fruiting body formation (Fig. 4B and C). Due to the overall delayed developmental programme of *atg7-*, its fruiting bodies started to form about 7 h later than in the Ax2 parent.

### 3.3 Induction of post-aggregative genes by cAMP in *atg7-*

**Fig. 5:**
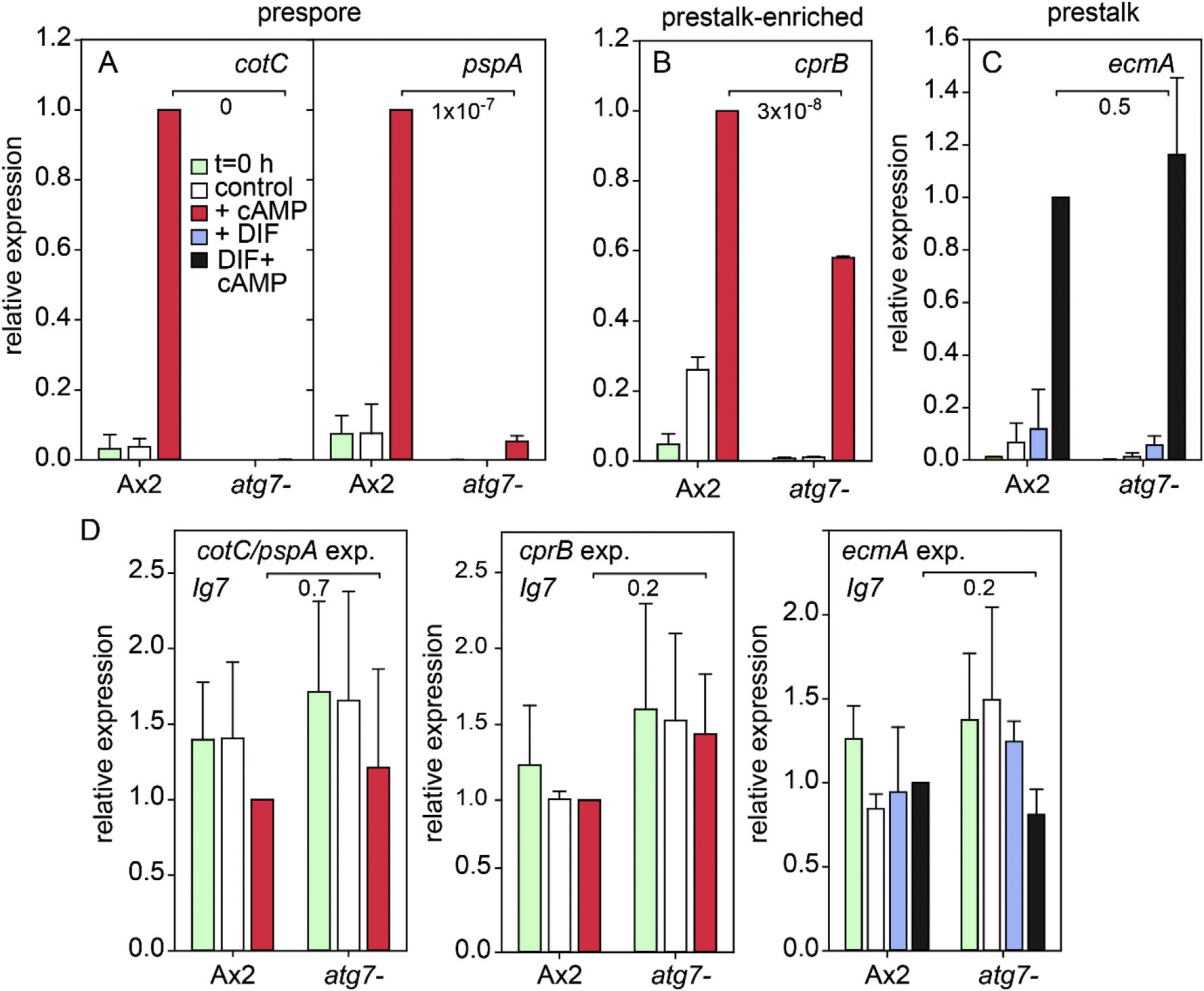
**Induction of cell type-specific genes by cAMP in *atg7-***. Ax2 and *atg7-* cells were developed into loose mounds (overnight at 12 °C) for prespore and *ecmA* induction or starved overnight at 4 °C for *cprB* induction. Aggregates were dissociated and cells were shaken in suspension with or without 1 mM cAMP and/or 100 nM DIF-1 as indicated. After 4 h, RNA was isolated and transcript levels were analysed by RT-qPCR, using primers specific for *cotC* and *pspA* (A), *cprB* (B) and *ecmA* (C). The RNAs from all different experiments were also probed with the constitutively expressed gene Ig7 (D). Data are expressed relative to expression in Ax2 in the presence of cAMP. Means and SD of three experiments, assayed with technical duplicates are presented. P-values of t-tests comparing cAMP-induced levels of the different genes between Ax2 and *atg7-* are shown underneath large brackets.

Prespore differentiation requires cAMP in the micromolar range acting on cell surface receptors (cARs) (Schaap and Van Driel, 1985; Wang et al., 1988a), and for many prespore genes, also intracellular cAMP acting on PKA (Hopper and Williams, 1994). Very low *cotC* expression in *atg7-* slugs (Fig. 4) prompted us to examine whether prespore gene induction by cAMP was impaired. Transcripts of the prespore genes *cotC* and *pspA,* but not of the constitutively expressed gene *Ig7,* strongly increased after 4 h of incubation with 1 mM cAMP in differentiation competent Ax2 cells, but remained low in competent *atg7-* cells and in the absence of cAMP (Fig. 5A,D). To assess whether defective cAMP induction of *atg7-* cells was specific to prespore genes, we also tested the prestalk gene *ecmA* and the prestalk-enriched gene *cprB* (CP2). *CprB* requires only cAMP for induction (Pears et al., 1985) and was induced in both Ax2 and *atg7-* cells, albeit that induction in *atg7-* was 40% lower than in Ax2 (Fig. 5B). *EcmA* is reported to require DIF-1 in addition to cAMP (Berks and Kay, 1990), and while effects of DIF-1 alone on *ecmA* induction were weak (Fig. 5C), induction by cAMP plus DIF-1 was high in both Ax2 and *atg7-* cells. These experiments show that *atg7-* cells are specifically defective in cAMP induction of prespore gene expression.

### 3.4 Is defective sporulation the result of reduced autophagy in *atg7-*?

**Fig. 6:**
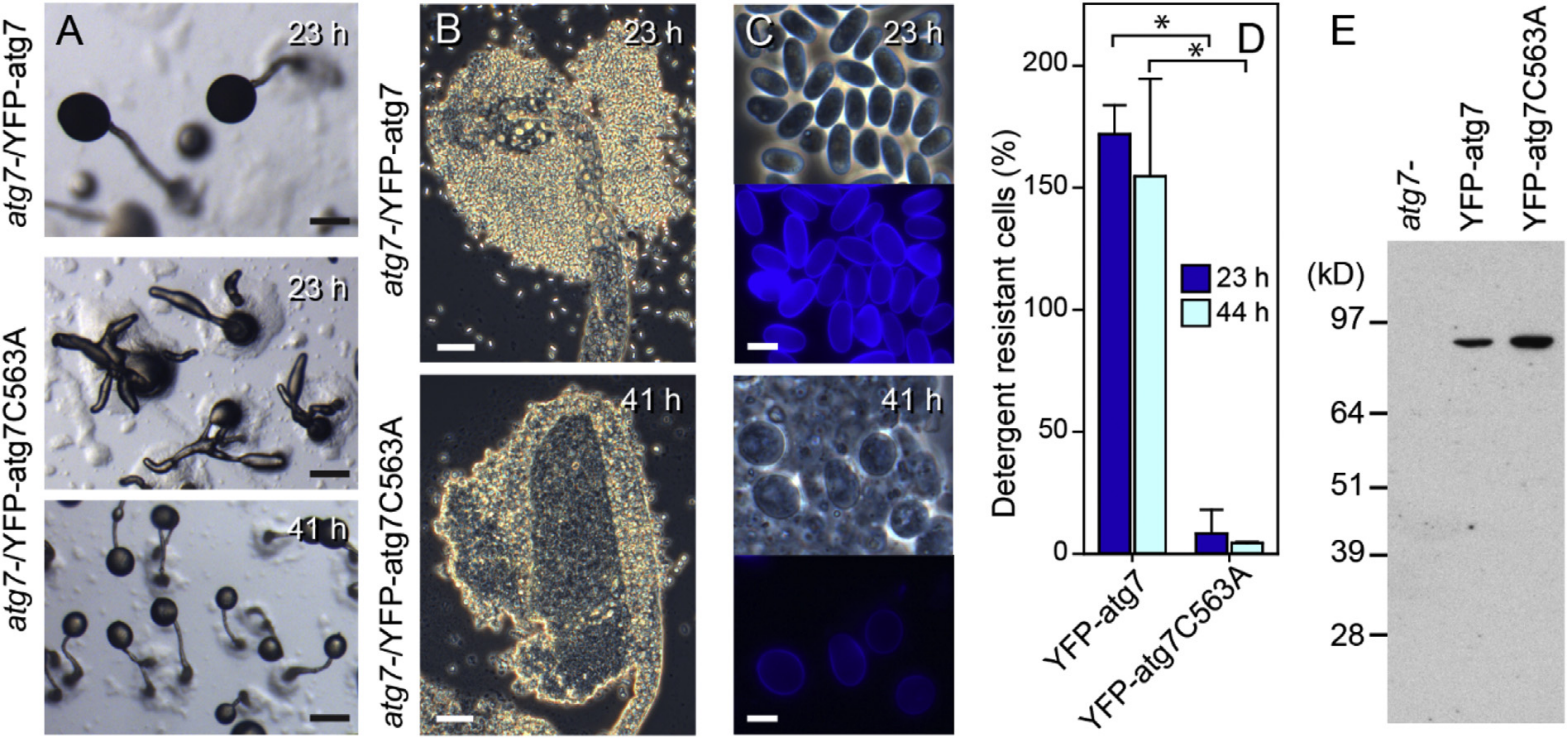
**Complementation of *atg7-* with atg7 and atg7-C563A**. *Atg7-* cells were transformed with a fusion construct of the actin15 promoter and the YFP and *atg7* coding sequences (act15p-YFP-atg7) or with act15p-YFP-atg7 harbouring a Cys563 to Ala mutation that deletes the Atg7 E1-like ligase activity. A,B. Cells were developed for 23 h and 41 h and structures were photographed *in situ* (A) or squashed below a coverslip (B). C. Cells from the spore head were also stained with 0.002% Calcofluor. Bars in A, B and C equal 200, 40 and 5 μm, respectively. D. The percentage of detergent resistant spores formed from a known number of plated cells was determined as described in the legend to Table 1, with spores harvested from both 23 ​h and 44 ​h fruiting bodies. *: significantly different, P ​< ​0.01. E. Lysates from *atg7-*, *atg7-*/act15p-YFP-atg7 and *atg7-*/act15p-YFP-atg7C563A cells were size-fractionated by SDS-PAGE and Western blots were probed with anti-GFP antibodies to visualize the YFP fusion proteins.

The requirement of Atg7 for cAMP induction of prespore gene expression suggests that autophagy is required for gene regulation. However, a role for Atg7, independent of autophagy, was shown in starving mouse fibroblasts, where binding of Atg7 to the p53 tumour suppressor was required for normal cell cycle arrest. This effect did not require the E1-like enzyme activity of Atg7, which mediates its role in autophagy (Lee et al., 2012). To assess whether the effects of loss of Atg7 in *Dictyostelium* are independent from its role in autophagy, we complemented the *atg7-* mutant with both intact Atg7 and Atg7 harbouring a Cys563 to Ala mutation, which abolishes its E1-like enzyme activity. Fig. 6 shows that only intact Atg7 restores normal fruiting body formation and sporulation in the *atg7-* mutants, indicating that the E1-like enzyme activity of Atg7 is required for sporulation.

**Fig. 7:**
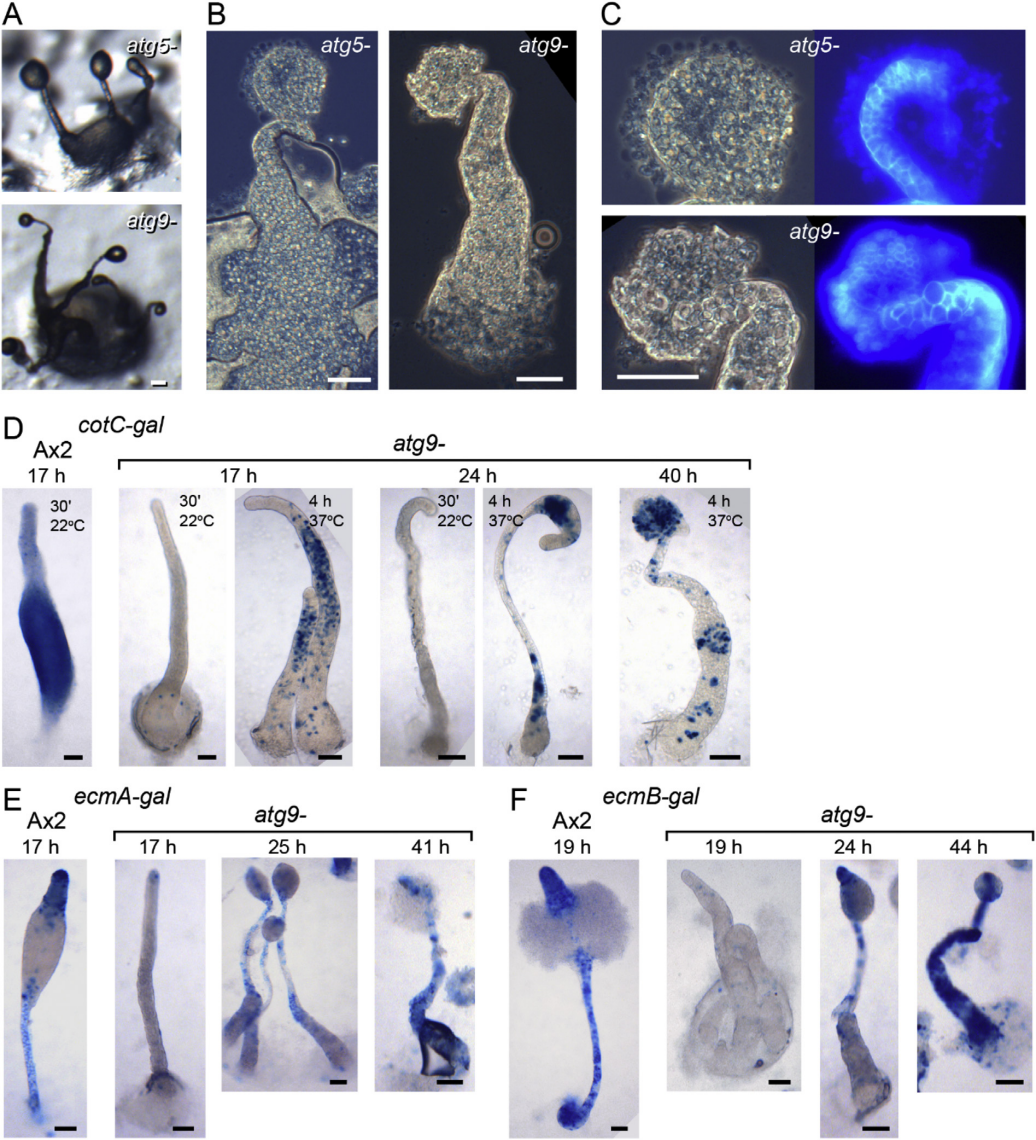
**Developmental phenotypes of *atg5-* and *atg9-* in an Ax2 background**. *A-C. Developing structures. Atg5-* and *atg9-* cells were developed on non-nutrient agar and terminal fruiting bodies were photographed *in situ* (A), or stained with Calcofluor, squashed under a coverslip and photographed under phase contrast (B, C left panel) or epifluorescence (C, right panel). Bar: 50 μm. *D-F. Cell-type specific gene expression.* Ax2 and *atg9-* cells, transformed with *cotC-gal* (D), *ecmA-gal* (E) and *ecmB-gal* (F) were incubated on dialysis membrane supported by non-nutrient agar for the time periods shown above the images. Structures were fixed and stained with X-gal for 30 min at 22 °C for all structures, and also for 4 h at 37 °C for some *atg9-/cotC-gal* structures as shown inside the images. Bar: 50 μm.

Several mutants in autophagy genes, such as *atg5*, *atg6*, *atg8* and *atg9* show a multi-tipped phenotype and spore defects (Otto et al., 2003, 2004; Tung et al., 2010). To assess whether specific defects in prespore gene induction are a general feature of autophagy mutants, we analysed the phenotype of *atg5-* and *atg9-* mutants in greater detail. Atg5 acts as a substrate in the first of the two steps of the ubiquitin-like system to conjugate Atg8 onto autophagosomes where Atg7 acts like an E1 enzyme, whereas Atg9 is required for recruiting lipid membrane in autophagosome formation (Lamb and Tooze, 2016). We investigated cell differentiation in an existing *atg9-* and recreated *atg5-* mutant (Fig. 1). During development on agar, *atg9-* and *atg5-* showed similar fruiting body defects as *atg7-,* with virtually absent spore formation, a relatively normal stalk with fully vacuolated and cellulose-encapsulated stalk cells and a large mass of basal cells that eventually also vacuolated (Fig. 7A–C). Similar to *atg7-*, *cotC-gal* expression was very low in *atg9-* and confined to a small region of the sorogens (Fig. 7D). Both *ecmA-gal* and *ecmB-gal* were expressed at similar levels in the primary stalk of developing fruiting bodies of Ax2 and *atg9-* (Fig. 7E and F). As was the case for *atg7-*, the enlarged bases of the *atg9-* fruiting bodies only expressed *ecmA-gal* and *ecmB-gal* very late in development.

**Fig. 8:**
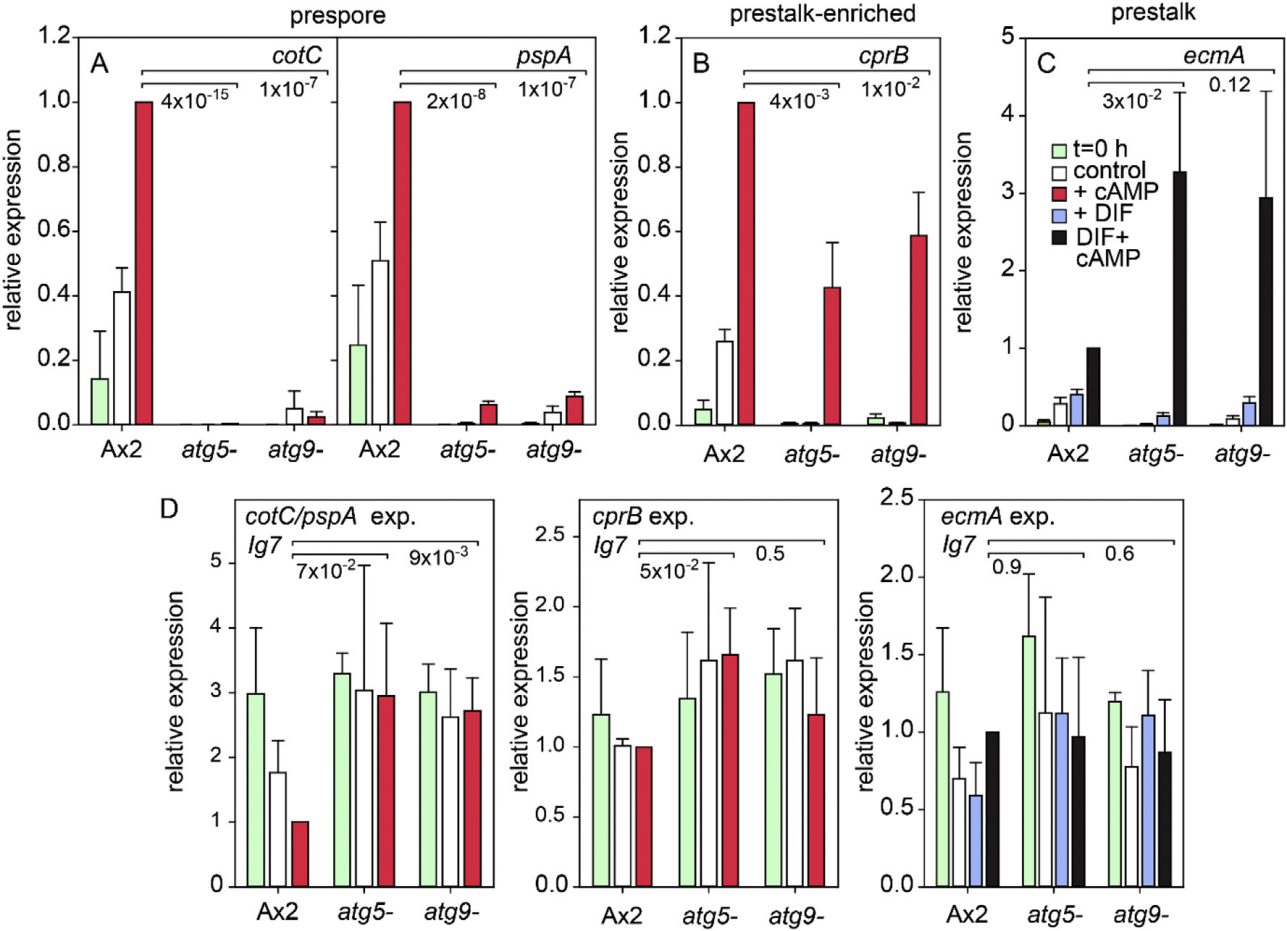
**Induction of cell type-specific genes by cAMP in *atg5-* and *atg9-***. Ax2, *atg5-* and *atg9-* cells were developed to differentiation competence and treated with cAMP and/or DIF-1 as described in the legend to Fig. 5, and transcript levels of the indicated genes and the constitutively expressed gene Ig7 were analysed by RT-qPCR. Means and SD of three experiments, assayed with technical duplicates. P-values of t-tests comparing cAMP or cAMP ​+ ​DIF-induced levels of the different genes between Ax2 and *atg5-* or *atg9-* are shown underneath large brackets.

cAMP induction of the prespore genes *cotC* and *pspA* was also absent from the *atg5-* and *atg9-* mutants, while *cprB* induction was 50% reduced. *EcmA* induction by cAMP and DIF was variable between experiments, but higher than in Ax2 (Fig. 8). Overall, these mutations in different aspects of the autophagy pathway all yielded the same phenotypic defects as *atg7-*, indicating that it is autophagy itself that is required for cAMP induction of prespore gene expression.

### 3.5 How does autophagy interact with cAMP induction of prespore gene expression?

The requirement of autophagy for cAMP-induced prespore expression could either result from i. cells not being competent to detect and process the cAMP signal, or ii. from autophagy being part of the cAMP signal transduction pathway by either producing a stimulator or degrading an inhibitor of prespore gene expression.

**Fig. 9:**
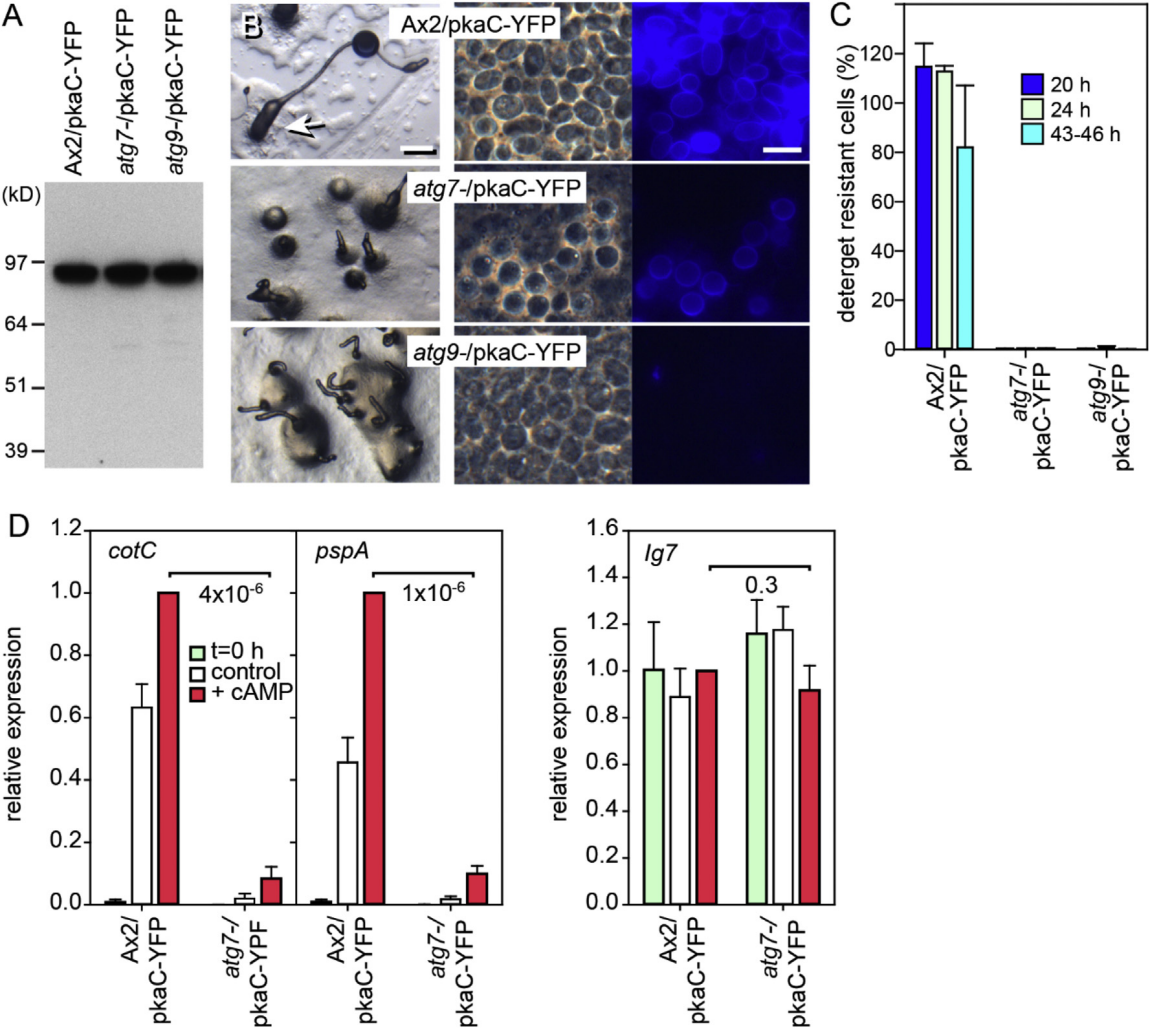
**Effect of PKA-C overexpression**. A. Ax2, *atg7-* and *atg9-* cells were transformed with act15p-pkaC-YFP and expression of pkaC-YFP was analysed by Western blot using anti-GFP antibody. B. Transformed cells were developed on agar for 19 h and photographed *in situ* (left panels, bar: 200 μm) or submerged in a droplet of 0.002% calcofluor and squashed under a coverslip (right panels, bar: 10 μm). The image of the Ax2/pkaC-YFP fruiting body was generated by focus stacking using Auto-Montage (http://www.syncroscopy.com). The large cell mass at its base (arrow) also contained spores (right panels). C. The percentage of detergent resistant spores formed from a known number of plated cells was determined as described in the legend to Table 3, with spores harvested from both 20, 24 h and 43–46 h fruiting bodies. Spore percentages between Ax2 and *atg7-* or *atg9-* transformants were significantly different, when compared pairwise at each time point, with P-values <0.01. D. Ax2 and *atg7-* cells transformed with act15p-pkaC-YFP were developed into loose mounds, dissociated and incubated for 4 h with or without 1 mM cAMP. RNA was isolated and transcript levels were analysed by RT-qPCR, using primers specific for the prespore genes *cotC* and *pspA* and the constitutively expressed gene Ig7. Data are expressed relative to expression in cAMP treated Ax2/pkaC-YFP cells. Means and SD of three experiments, assayed with technical duplicates are presented. P-values of t-tests comparing cAMP-induced levels between Ax2 and *atg7-*derived cells are shown in the panels.

In addition to cAMP activation of cAMP receptors (cARs), cAMP activation of protein kinase A (PKA) is required for expression of many prespore genes and for spore maturation (Hopper et al., 1993), with precocious sporulation being induced by overexpression of the PKA catalytic subunit, PkaC (Mann et al., 1994). Because cARs also activate adenylate cyclase A (Theibert and Devreotes, 1986), PKA can potentially act downstream of cARs. We therefore examined whether PkaC overexpression can restore sporulation in *atg7-* and *atg9-.* Fig. 9 shows that PkaC overexpression in Ax2 caused precocious maturation of many spores at the sorogen base, before the prespore mass had ascended the stalk. However, no viable spores were formed in *atg7-* and *atg9-* overexpressing PkaC. To test whether the failure of PkaC to rescue sporulation in *atg7-* already acted at the stage of prespore gene induction, we compared cAMP induction of the prespore genes *cotC* and *pspA* between Ax2 and *atg7-* cells, both overexpressing pkaC-YFP. Fig. 9D shows that levels of *cotC* and *pspA* expression in response to cAMP stimulation remained very low in the *atg7-*/PkaC-YFP cells, indicating the pkaC does not act downstream of Atg7.

**Fig. 10:**
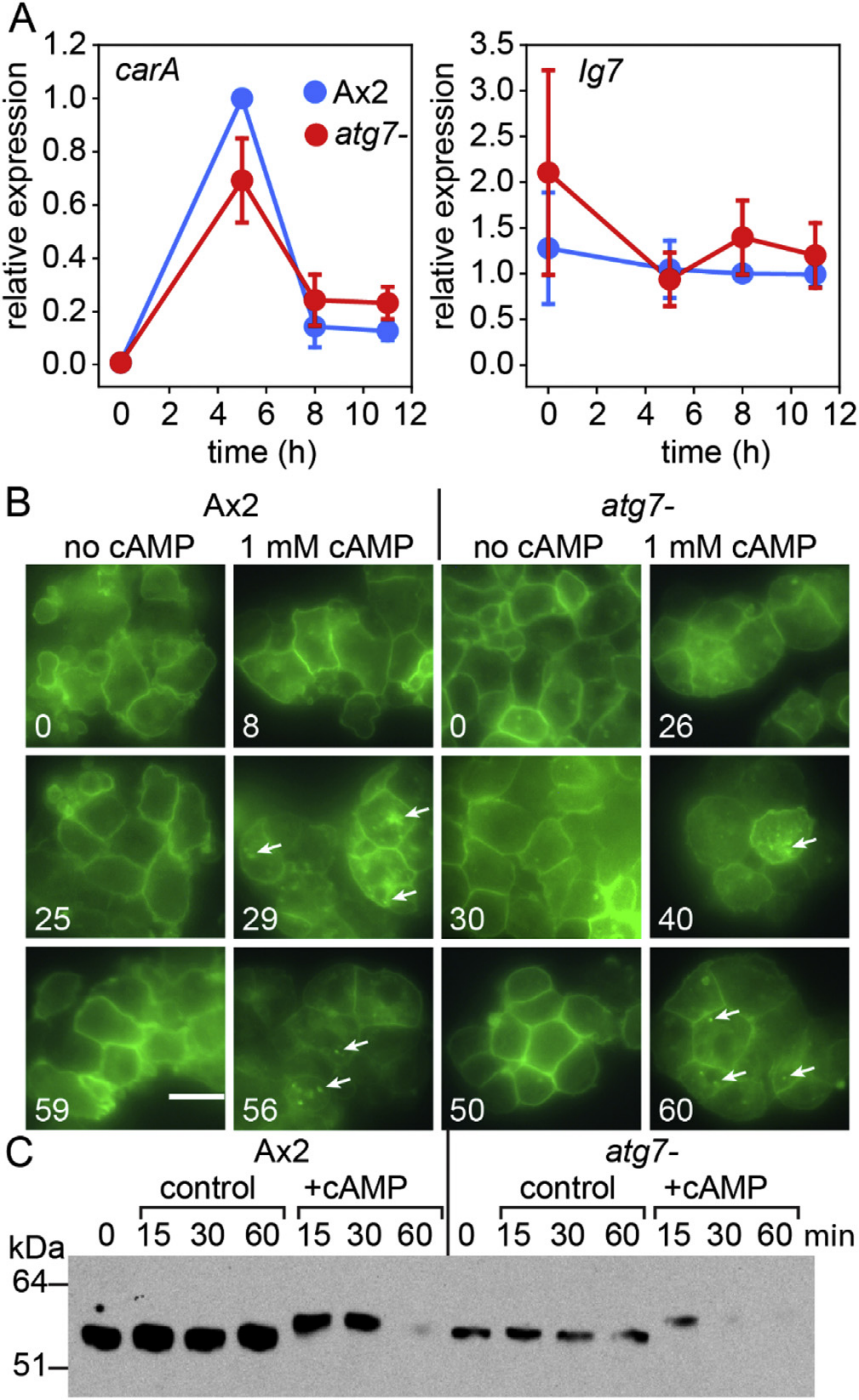
**cAMP receptor levels, internalization and phosphorylation in *atg7-***. *A. cAR gene expression.* Ax2 and *atg7-* cells were developed at 22 °C on non-nutrient agar. RNA was extracted at the indicated time periods and analysed for *carA* and *Ig7* expression by RT-qPCR. *B. Internalization.* Dissociated loose mound cells of Ax2 and *atg7-,* transformed with A15-cAR1-GFP (Xiao et al., 1997) were incubated in the presence or absence of 1 mM cAMP and photographed under epifluorescence at the indicated time points in minutes. Arrows highlight patches of internalised cAR1-GFP. Bar: 10 μm. *C. cAR1 band-shift.* At the indicated time points after cAMP addition, cells were lysed in SDS sample buffer and analysed by Western blot, using anti-GFP antibody.

The cAMP receptors cAR1, 2 and 3 can largely complement each other's function in mediating cAMP-induced prespore gene expression. However, cAR1, the only cAR, which, like prespore induction, is inhibited by adenosine, most likely mediates this process (Verkerke-VanWijk et al., 1998). We first tested whether expression of the cAR1 gene *carA* was defective in the *atg7-* mutant. Fig. 10A shows that *carA* transcript levels were only marginally reduced in *atg7-* during the first 12 h of development, indicating that its lack of cAMP-induced prespore gene expression is not due to lack of cAR1. During persistent stimulation with micromolar cAMP, cAR1 receptors are endocytosed and eventually degraded (Wang et al., 1988b). Autophagy was also reported to promote endocytosis of membrane receptors (Shin et al., 2016; Xu et al., 2016). We therefore tested whether Atg7 was required for internalization of cAR1, which could be part of the cAMP pathway activating prespore genes. We transformed both Ax2 and *atg7-* cells with a cAR1-GFP fusion construct, expressed from the constitutive actin 15 promoter (Xiao et al., 1997) and observed cAR1-GFP localization in the absence and presence of cAMP. Fig. 10B shows that cAR1-GFP remains mostly membrane localized in both Ax2 and *atg7-* in the absence of cAMP. Upon cAMP stimulation cAR1-GFP patches appear inside both Ax2 and *atg7-* cells, while staining at the cell periphery decreases. Upon persistent stimulation with cAMP, cAR1 is also phosphorylated, which causes a mobility shift on SDS-PAGE, followed by protein degradation (Vaughan and Devreotes, 1988). Fig. 10C shows that both Ax2 and *atg7-*, transformed with cAR1-GFP showed a mobility shift and subsequent degradation of the cAR1-GFP. It is therefore unlikely that autophagy stimulates prespore gene expression by causing cAR1 internalization.

**Fig. 11:**
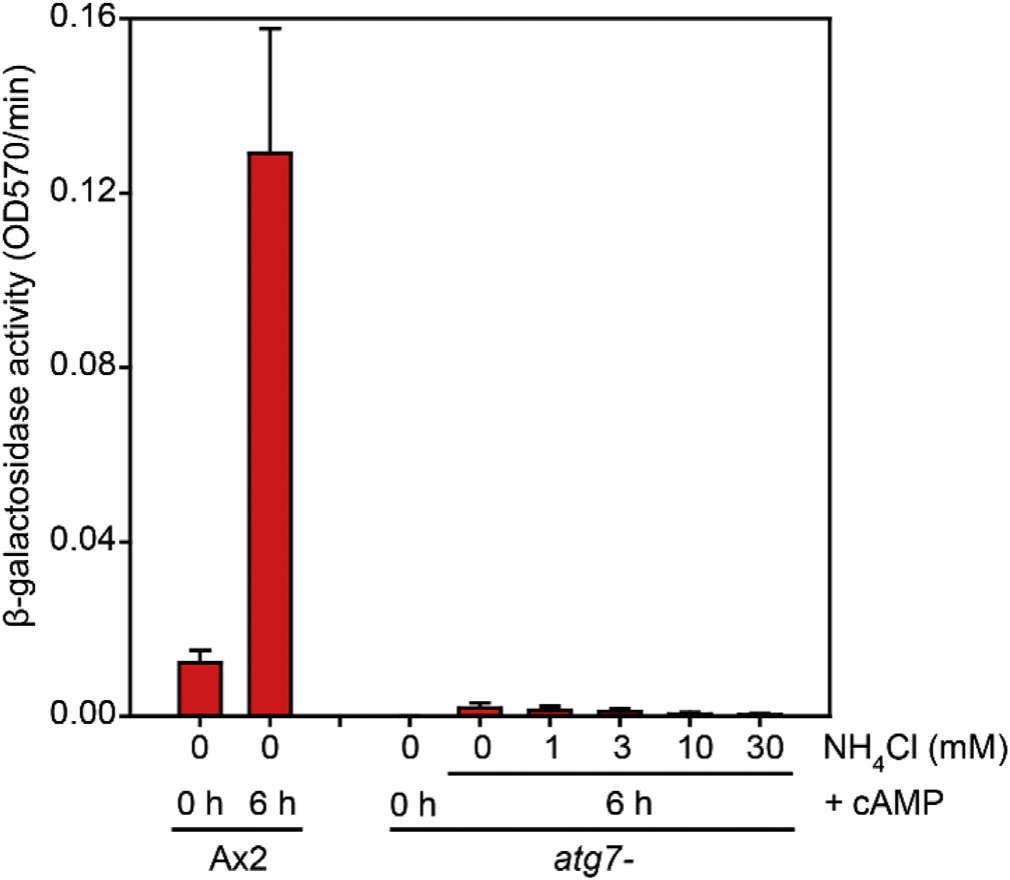
**Effect of ammonia on cAMP induction of *cotC-gal***. Ax2 and *atg7-* cells were transformed with *cotC-gal* and developed into loose mounds. Cells were then dissociated in 20 mM potassium phosphate pH 7.4 containing 1 mM MgCl_2_ and incubated with cAMP and increasing concentrations of NH_4_Cl for 6 h. After cell lysis, expression of *cotC-gal* was analysed with a spectrophotometric β-galactosidase assay. Means and SD of two experiments performed in duplicate. Values for cAMP-treated Ax2 cells were significantly higher than for cAMP-treated *atg7-* cells at each of the NH_4_Cl concentrations at P < 0.005.

Protein degradation by autophagy yields ammonia, which was reported to promote spore differentiation (Bradbury and Gross, 1989; Gross et al., 1983). To investigate whether lack of ammonia caused defective cAMP-induced prespore gene expression in autophagy mutants, we treated *atg7-/cotC-gal* cells with cAMP and increasing ammonia concentrations. However, there was no restoration of *cotC-gal* induction by ammonia (Fig. 11).

**Fig. 12:**
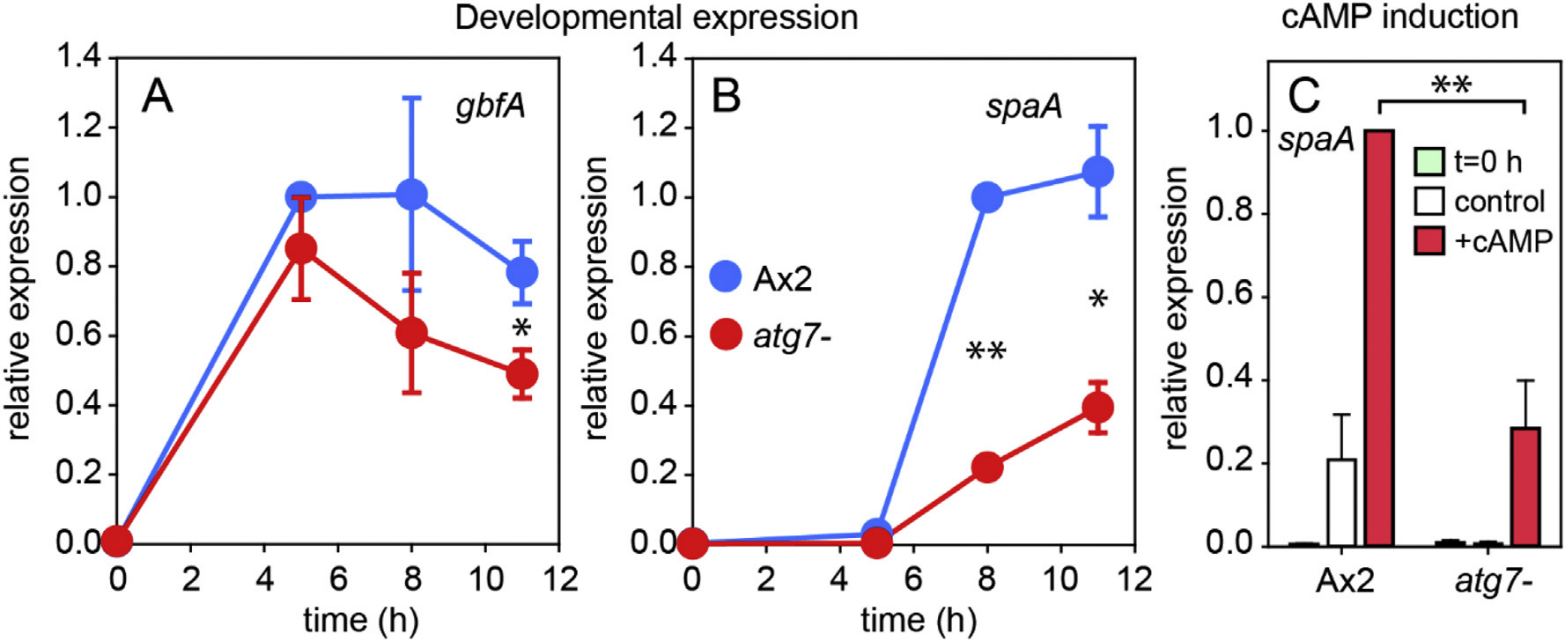
**Expression of transcription factors in the *atg7-* mutant**. A,B. *Developmental regulation*. The RNAs isolated for analysis of *carA* expression in Fig. 6 were used to study *gbfA* (A) and *spaA* (B) expression using *spaA* and *gbfA* specific primers (Table 2). C. *cAMP induction.* The RNAs isolated for analysis of *cprB* induction in Fig. 5 were here analysed for *spaA* induction. Significant differences in expression between Ax2 and *atg7-* at the same developmental time (A,B) or after induction with cAMP (C) are marked with * for 0.01 ​< ​P ​< ​0.05 and ** for P ​< ​10^−5^.

We finally tested how loss of autophagy affected two transcription factors with roles in prespore gene expression. The transcription factor SpaA acts downstream of PKA to induce expression of some prespore genes and spore maturation (Yamada et al., 2018). GbfA was isolated as a protein binding to G-rich motifs in the prestalk gene *cprB*, and to be required for cAMP induction of this gene. Subsequent studies showed that it also interacted with G-rich motifs in prespore genes and was required for their cAMP-inducibility (Hjorth et al., 1989; Powell-Coffman et al., 1994; Schnitzler et al., 1994). Expression of *gbfA* is about 30% reduced in *atg7-* cells (Fig. 12A), which can account for the ∼40% reduction of cAMP induction of *cprB*, but not the complete loss of *cotC* and *pspA* induction (Fig. 5). *SpaA* expression is about 60% reduced in *atg7-* (Fig. 12B), also not enough to account for complete loss of *cotC* induction. *SpaA* is only expressed in prespore and spore cells and we tested whether it was itself induced by cAMP. This was the case (Fig. 12C) with induction being ∼70% reduced in *atg7-* cells, in agreement with its 60% reduced developmental expression. Overall, the effects of loss of Atg7 on *spaA* and *gbfA* expression are insufficient to account for the complete lack of prespore induction in *atg7-* cells.

## 4 Discussion

### 4.1 Disruption of autophagy prevents cAMP induction of prespore differentiation

A screen for mutants defective in spore differentiation identified Atg7, a component of the autophagy pathway as being essential for this process (Fig. 2). Further analysis showed that the *atg7-* mutant was specifically impaired in cAMP induction of prespore gene expression, but not in cAMP-induction of different classes of prestalk genes and in stalk cell differentiation (Fig. 5). Closer investigation of mutants lacking Atg5 and Atg9 revealed that they were also specifically defective in cAMP-induced prespore gene expression, indicating that the defect in *atg7-* mutants was due to loss of autophagy and not to a role of Atg7, unrelated to autophagy.

Because *Dictyostelium* cells go through multicellular development while starving, loss of autophagy, which essentially deprives cells of metabolites and sources of energy can be expected to interfere with several developmental processes, which is evident from the abnormal multi-tipped morphology of autophagy mutants and effects on expression of many classes of genes (Calvo-Garrido et al., 2010; Mesquita et al., 2017) (Fischer et al., 2019). Deleterious effects of loss of Atg7 and other autophagy proteins on spore formation was reported before (Otto et al., 2003, 2004; Tung et al., 2010). However, these studies focussed on resolving the autophagy pathway and did not study sporulation in detail. Loss of autophagy also prevents production of the spore maturation inducing peptide SDF-2 (Cabral et al., 2010), but unlike the cell-autonomous defect in prespore gene expression caused by loss of autophagy reported here, this is a non-cell autonomous defect that acts much later in development. Since spore differentiation requires a considerable investment in building materials for construction of the multi-layered spore wall, defective sporulation in autophagy mutants is to be expected. However, the early and specific effect of autophagy loss on cAMP-induced prespore gene expression is enigmatic.

### 4.2 Role of autophagy in stalk cell differentiation

The lack of effect of loss of autophagy genes on stalk cell differentiation was unexpected since autolysosome formation occurs much more prominently in prestalk than prespore cells, while fusion of autolysosomes is considered to cause formation of the large central vacuole of the stalk cells (Schaap, 1983; Uchikawa et al., 2011). Furthermore, loss of two other autophagy genes *atg1* or *vmp1* prevent cells from differentiating into stalk cells *in vitro* in response to DIF-1, an inducer of stalk cell differentiation (Calvo-Garrido and Escalante, 2010; Kosta et al., 2004). The *atg7-* mutant also showed normal DIF-induced stalk cell differentiation *in vitro* (Fig. 3), and, like *atg5-* and *atg9-* , formed somewhat disorganized, but otherwise normal stalks *in vivo* (Fig. 2, Fig. 7), and this was also reported for *vps13-*mutants (Muñoz-Braceras et al., 2015).

The relatively normal stalk cell differentiation in *atg7-*, *atg5-, atg9-, vps13-* and likely all other autophagy mutants that still manage to form fruiting structures could be a consequence of the fact that unlike *atg1*, these genes act more downstream in the autophagy pathway and might not inhibit autophagy altogether. Also non-canonical forms of autophagy that do not require Atg5 or Atg7 have been described (Arakawa et al., 2017; Dupont et al., 2017). Another explanation could be that both Atg1 and Vmp1 have other roles that prevent stalk cell differentiation specifically. Both *atg1-* and *vmp1-* cells show early developmental arrest (Calvo-Garrido et al., 2008; Otto et al., 2004). In addition to autophagy, Vmp1 is also involved in organelle biogenesis, contractile vacuole function and protein secretion (Calvo-Garrido et al., 2008), and interference with these functions may preclude normal developmental progression. Apart from initiating phagophore formation, Atg1 regulates the activity of transketolase, an enzyme in the pentose phosphate pathway (Mesquita et al., 2015).

However, if autophagy does not cause stalk cell vacuolization, as the phenotypes of most autophagy mutants, except *atg1-* and *vmp1-*, suggest, than what does? One possibility is that the stalk vacuole is more akin to the large central vacuole of plants or fungi that fulfils a range of functions. While both the plant and fungal vacuoles also fuse with autophagosomes during the normal progression of autophagy (Yoshimoto and Ohsumi, 2018), the biogenesis of these vacuoles is not dependent on autophagy. *Dictyostelium* may have as yet unnoticed protovacuoles that normally own much of their size increase to fusion with autophagosomes, but can also inflate in their own right.

### 4.3 Elimination of putative prespore pathway components that are affected by autophagy

While a pleiotropic effect of autophagy on the formation of normal viable spores is to be expected, direct involvement of non-specific digestion of cell contents on a specific cAMP signal transduction pathway is difficult to explain. The sporulation defect of *atg5-*, *atg7-* and *atg9-* mutants is cell-autonomous (Otto et al., 2003, 2004) (Table 3), which excludes that the defect is caused by the absence of signal molecules or materials produced by e.g. the prestalk cells. The cAMP receptors (cARs) that mediate cAMP-induction of prespore genes are expressed at normal levels in *atg7-* cells (Fig. 10A), while cAMP-induced prestalk gene expression, which occurs at the same developmental stage as prespore gene induction is not impaired (Fig. 5, Fig. 8). This indicates that lack of autophagy does not generally interfere with the acquisition of differentiation competence.

In addition to extracellular cAMP acting on cARs, intracellular cAMP acting on PKA is also required for expression of some prespore genes, such as *cotC,* and for terminal spore maturation (Hopper et al., 1993). *CotC* expression requires the transcription factors CudA and SpaA, which likely act downstream of PKA (Yamada et al., 2008, 2018). However, PKA overexpression neither rescued cAMP induction of prespore gene expression in *atg7-*, nor sporulation in either the *atg7-* or *atg9-* mutant (Fig. 9), indicating that defective autophagy does not act by preventing PKA activation. This is further substantiated by the observation that cAMP induction of the prespore gene *pspA*, which does not require PKA, SpaA or CudA for expression (Hopper et al., 1993; Yamada et al., 2018) is also lacking in the *atg7-* mutant (Fig. 5, Fig. 8). The transcription factor GbfA is required for cAR regulated expression of prespore and prestalk genes, particularly *cprB* (Hjorth et al., 1989; Powell-Coffman et al., 1994; Schnitzler et al., 1994). While *gbfA* expression is ∼40% reduced in *atg7*- (Fig. 12A), this reduction can account for the similarly reduced *cprB* expression, but not for the complete absence of prespore gene expression (Fig. 5, Fig. 8).

cAR1 phosphorylation and endocytosis accompany induction of prespore gene expression by micromolar cAMP and could potentially mediate this process (Vaughan and Devreotes, 1988; Wang et al., 1988b). However, neither cAR1 phosphorylation nor internalization were impaired in the *atg7-* mutant (Fig. 10). For autophagy to mediate cAMP-induced prespore gene expression, autophagy itself should be activated by cAMP. However, autophagic vesicles are actually down-regulated in prespore cells (Schaap, 1983). This leaves the possibilities that autophagy either produces a catabolite that is specifically required for the cAMP pathway or eliminates a pathway specific inhibitor. An obvious catabolite of protein degradation is ammonia, which is known to promote spore and inhibit *Dictyostelium* stalk cell differentiation (Bradbury and Gross, 1989; Gross et al., 1983) (Wang and Schaap, 1989). However, we could not restore cAMP induction in the *atg7-* mutant by co-incubation with ammonia (Fig. 11).

No further pathway components or inhibitors thereof are known, ending our options to identify the nature of the interaction between autophagy and prespore gene expression by a biased approach. Our current forward genetic strategy to identify sporulation genes, or a genetic screen for a suppressor of the *atg7-* phenotype is more likely to identify the prespore pathway components that are affected by loss of autophagy.
